# Supplementary material for: A spatial perturbation framework to validate implantation of the epileptogenic zone
Source: Nat Commun. 2024 Jun 19;15:5253. doi: 10.1038/s41467-024-49470-z (PMC11187199; doi:10.1038/s41467-024-49470-z)
Supplement: Supplementary file 1 — Supplementary Information [file 41467_2024_49470_MOESM1_ESM.pdf]

Supplementary Method S1: Biomarker extraction

IED and ripple rates were previously shown to localize the EZ and were used to validate the use of the IED-γ rate [1-3]. IEDs and ripples were automatically detected using previously validated detectors [4,5]. The IED rates, IED$-\gamma$, and ripple rates were then computed and reported as rates per minute. Fast ripples (250-500Hz) were not considered, as the validation cohort mainly contained SEEG data with sampling rate of 512 Hz.

The IED-γ detection was employed in accordance with Thomas et al.'s publication [3] using the code available at the GitHub repository: https://github.com/Lab-Frauscher/Spike-Gamma. The following are the key steps:

1. Detect the IEDs using the Janca detector [4]: Firstly, the signal was downsampled to 200Hz, and the envelope of the bandpassed signal [10,60Hz] is computed. An IED is detected within a 5s moving window (80% overlap) if the upper percentile (i.e., $3.65 \times (\text{mode} + \text{median})$) of the distribution of the signal envelope computed from the 5s moving window, intersects with the signal envelope. The IED detections using the Janca detector were validated in previous studies [3,6,7].
2. Postprocessing: All detected IEDs that occurred within 300 ms in the same channel were ignored. IEDs were also ignored if the detections were made simultaneously in at least 50% of channels.
3. IED-γ detections: For each detected event, the IED onset and offsets were delineated after the signal was band-passed [0.3, 500Hz] for MNI data, and [0.3, 250Hz] for CHUGA (sampling rates of 2000 and 512 Hz, respectively) using a fourth order Butterworth filter. After the onset and offset was determined, the γ-activity [30-100Hz] is compared before the IED onset and after the IED offset using a 500ms window. An IED is classified as an IED-γ if the 500ms window preceding the onset of the IED contains significant γ-activity greater than two standard deviations with at least 3 cycles.

Supplementary Method S2: ROC curves and thresholds

The ROC curve was plotted as the true-positive rate (TPR) against the false-positive rate (FPR), which are calculated as follows:

$$\mathrm{FPR} = \frac{\mathrm{FP}}{\mathrm{FP} + \mathrm{TN}}; \quad \mathrm{TNR} = \frac{\mathrm{TP}}{\mathrm{TP} + \mathrm{FN}}$$

The plot is calculated by ranging a threshold and reporting the TPR and FPR for each threshold in the ROC curve. The optimal threshold was calculated by intersecting the ROC curve with the slope S of a straight line as performed in the MATLAB function `perfcurve`

$$S = \frac{|\mathrm{FP}|}{|\mathrm{FN}|} \times \frac{|\mathrm{P}|}{|\mathrm{N}|}$$

Where $|\mathrm{FP}|, |\mathrm{FN}|, |\mathrm{P}|,$ and $|\mathrm{N}|$ denote the number of false-positive (FP), false-negative (FN), positive and negative classifications. The area under the curve (AUC) is defined as the area under the ROC curve, which quantifies a model's classification performance. An ideal classifier would have an AUC of 1.00, and a very poor classifier would have an AUC of 0.50, equivalent to a coin flip.

Supplementary Method S3: K-means clustering analysis

Unsupervised clustering was performed to classify the implantation scheme without the need of surgical outcomes. The k-means clustering algorithm was executed using the MATLAB function `kmeans`.

Feature selection

Firstly, the features were chosen to maximize the separation and compactness of the clusters using Dunn's index as the appropriate measure[8]. For interpretability, we aimed to represent a three-dimensional feature space. A set of features $\mathbf{F} = \langle \mathbf{F}_1, \mathbf{F}_2, \mathbf{F}_3, \mathbf{F}_4 \rangle$ are calculated using quadrants Q_1, Q_2, Q_3, and Q_4 (see **Methods**). The Dunn's index is calculated for each feature combination defined as follows:

$$\mathrm{DI}_{k,\boldsymbol{F}} = \frac{\min\limits_{1 \le i < j = k} \delta\left(\mathbf{C}_{i,\mathbf{F}}, \mathbf{C}_{j,\mathbf{F}}\right)}{\max\limits_{1 \le m \le k} \boldsymbol{\Delta}_{m,\mathbf{F}}}$$

Where $\mathbf{C}_{i,\mathbf{F}}$ represents the vector of features for a given feature combination $\mathbf{F}$ found in cluster i, where $i \in \{1,2,\ldots,k\}$ for k clusters. $\delta(\mathbf{C}_{i,\mathbf{F}}, \mathbf{C}_{j,\mathbf{F}})$ denotes the inter-cluster distance, which is the distance between the features in $\mathbf{C}_{i,F}$, and the features in $\mathbf{C}_{j,\mathbf{F}}$. $\boldsymbol{\Delta}_{\mathrm{m},\mathbf{F}}$ denotes the intra-cluster distances between two features in $\mathbf{C}_{k,\mathbf{F}}$, where $m \in \{1,2,\ldots,k\}$. The compactness is calculated in the denominator of $\mathrm{DI}_{k,\mathbf{F}}$, and the separability is calculated in the numerator of $\mathrm{DI}_{k,\mathbf{F}}$. Therefore, the larger the index, the more separable and compact the clusters are. We used the Euclidean distance in this study. Since we do not yet know the optimal number of clusters, we obtain the maximum Dunn's index for $k = 2,\ldots,10$.

$$\mathrm{DI}_k = \underset{\mathbf{F}}{\mathrm{argmax}}\left(\mathrm{DI}_{k,\mathbf{F}}\right)$$

The most frequent feature combination which has the maximum DI_k was found to be $\mathbf{F}_{\mathrm{opt}} = \langle \mathbf{F}_1, \mathbf{F}_2, \mathbf{F}_4 \rangle$, and is then chosen for subsequent analysis. A graphical representation of the index is shown in **Supplementary Fig. S12.** Dunn's index was computed using a third-party MATLAB code (https://www.mathworks.com/matlabcentral/fileexchange/27859-dunn-s-index). The Dunn's index for all combinations is shown in **Supplementary Table S4**.

Finding optimal clusters

We noticed that the DI_k for $k = 2,3,4$ are quite similar. Therefore, the next step is to find the optimal number of clusters k_{opt}. This is performed using the L-curve technique, which essentially finds a balanced trade-off between the number of clusters and the sum of distance error, which is calculated as follows:

$$e_d(k) = \sum_{j=1}^{k} \sum_{i=1}^{N_i} \left(\mathbf{C}_{i,\mathbf{F}_{\mathrm{opt}}} - \mathbf{c}_j\right)^2$$

Therefore, we aim to obtain the knee point of $e_d(k)$, where $\mathbf{c}_j$ is the centroid obtained using the k-means clustering algorithm. The knee point of $e_d(k)$ was calculated using a third-party MATLAB function `knee_pt` (https://www.mathworks.com/matlabcentral/fileexchange/35094-knee-point), obtaining $k_{\mathrm{opt}} = 2$ as the optimal number of clusters (see **Supplementary Fig. S8b**). We also determined the optimal number of clusters using the GAP statistic[10], and found that $k_{\mathrm{opt}} = 2$.

FIGURES

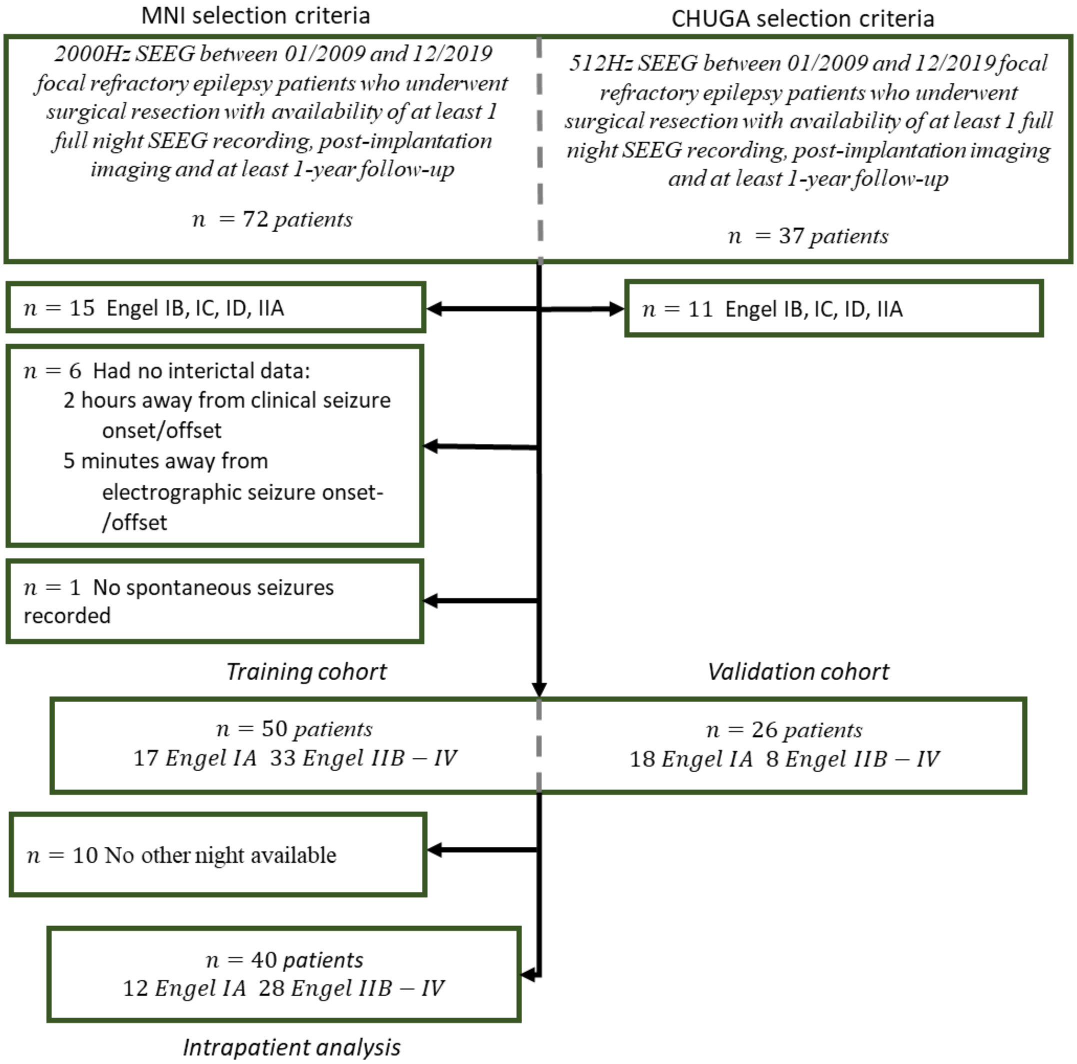

Supplementary *Figure S1: Patient selection flowchart.* *Out of the 72 patients, 50 patients have been considered for analysis as per the inclusion and exclusion criteria. Twenty-two patients were excluded due to the following reasons: patients classified as Engel IB-IIA (n=15); no available interictal segment as per the inclusion criteria (n=6); and the absence of spontaneous seizures recorded during presurgical investigation (n=1).*

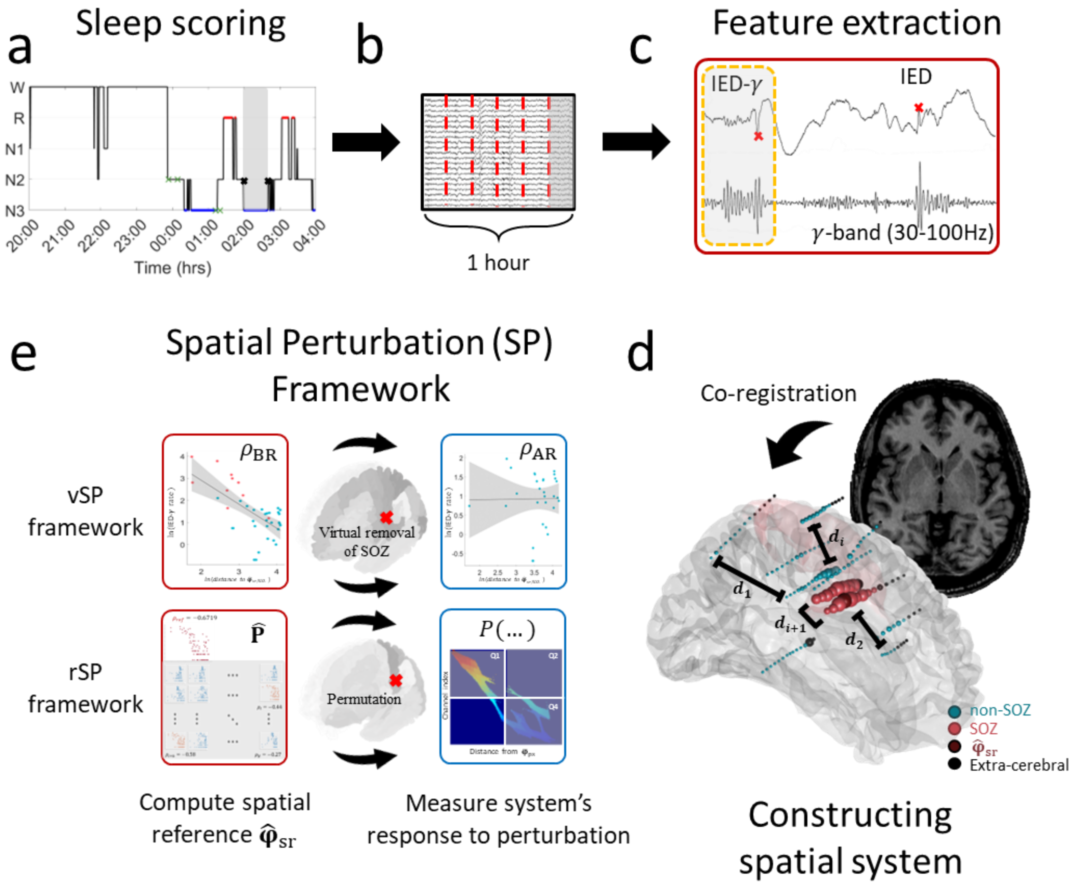

Supplementary Figure S2: Complete methodology pipeline used in this study. a *Sleep scoring using the SleepSEEG algorithm was applied to the overnight data* [11]*. Electrographic seizures marked with green crosses and the shaded region illustrates the selected continuous one-hour segment selected during NREM sleep.* ***b*** *10-minute segmentation of the complete hour.* ***c*** *IED-γ are detected by finding IEDs with significant gamma activity preceding its onset as performed in* Ren, et al. [12]*, and shown to localize the EZ with high specificity* [3]*.* ***c*** *SEEG channel coordinates are obtained by performing linear co-registration of post-implantation imaging with a template in normalized MNI space* [13]*. Extracerebral and white matter channels are subsequently removed, as well as channels exhibiting significant artifacts.* ***d*** *The SP framework is formulated by first constructing a spatial system, which couples IED-γ rates and their distance to a spatial reference. Two kinds of perturbation were applied in this study, and the change in the spatial coupling is quantified by computing the system's perturbation strength. Abbreviations: IED=interictal epileptiform discharge, BR=before removal, AR=after removal, SOZ=seizure onset zone, MNI=Montreal Neurological Institute, SP=spatial perturbation, vSP=virtual-removal SP, rSP=ranked SP,* $\hat{\varphi}_{sr}$ *=spatial reference.*

Perturbation strengths

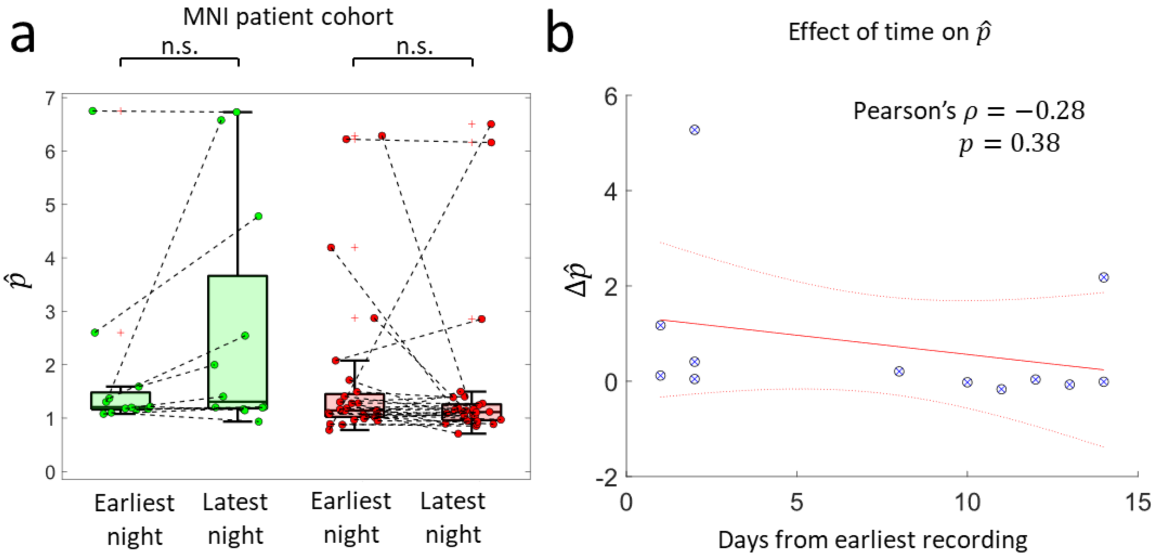

Supplementary Figure S3***: Perturbation strength is not influenced by the duration of implantation.*** ***a*** *There was no statistical difference in perturbation strength when an earlier night was considered in the seizure-free cohort (p=0.064;n=12; two-sided paired Wilcoxin signed rank test*) *and non-seizure-free cohort (p=0.25;n=30; two-sided paired Wilcoxin signed rank test*). ***b*** *There is no statistical difference in regard to the days after the earliest selected night with respect to the change in perturbation strength (p=0.38; permutation test*), *implying that the increase is not substantial. Source data are provided as a Source Data file. Abbreviations: MNI=Montreal Neurological Institute.*

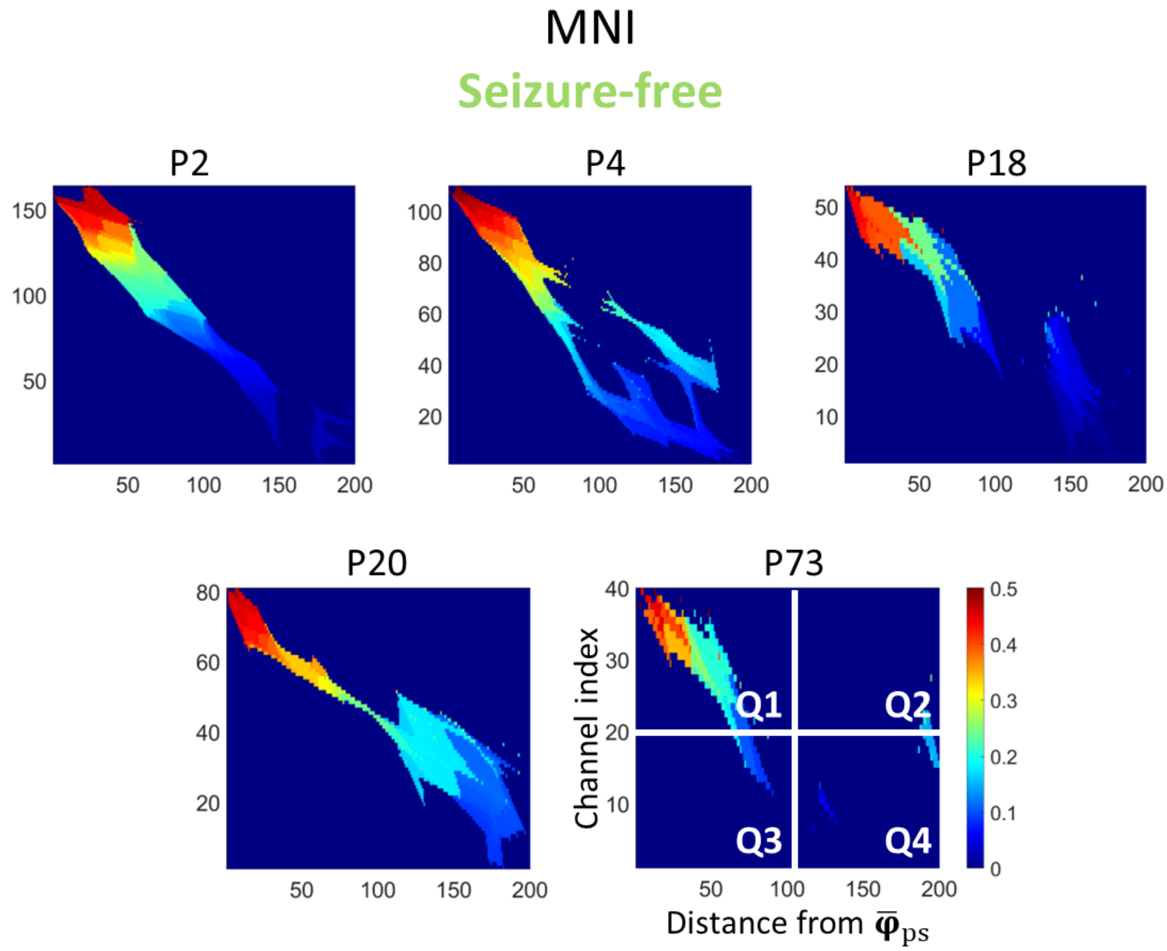

Supplementary Figure S4*: Examples of seizure-free SP maps from the MNI patient cohort.* *Five examples of the SP maps computed from seizure-free patients in the MNI cohort are shown to demonstrate the consistencies in the patterns described in **Figure 6** of the main manuscript. Abbreviations: MNI=Montreal Neurological Institute, Q=Quadrant, SP=Spatial perturbation.*

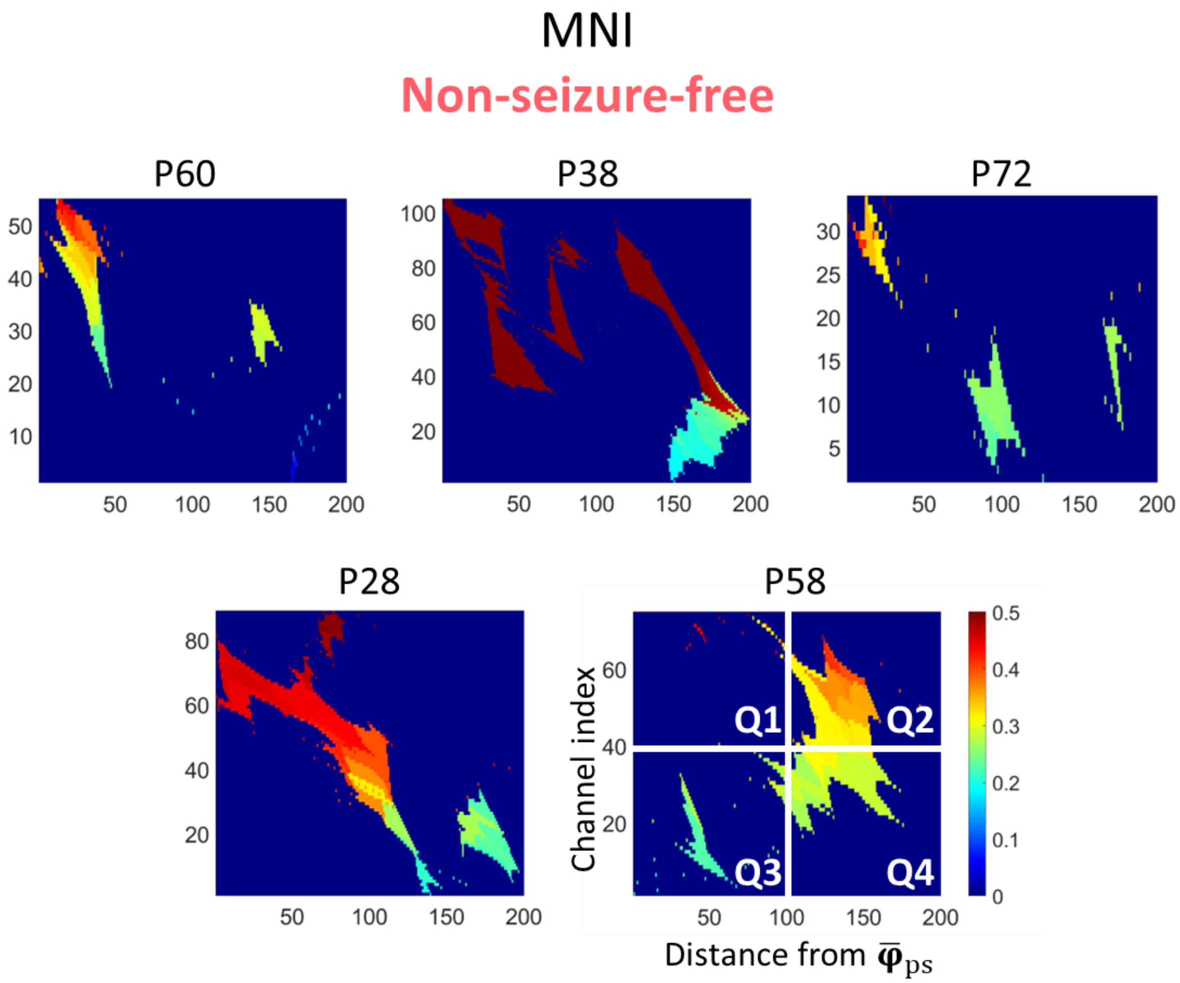

Supplementary Figure S5***: Examples of non-seizure-free SP maps from the MNI patient cohort.*** *Five examples of the SP maps computed from non-seizure-free patients in the MNI cohort are shown to demonstrate the consistencies in the patterns described in* ***Figure 6*** *of the main manuscript. Abbreviations: MNI=Montreal Neurological Institute, Q=Quadrant, SP=Spatial perturbation.*

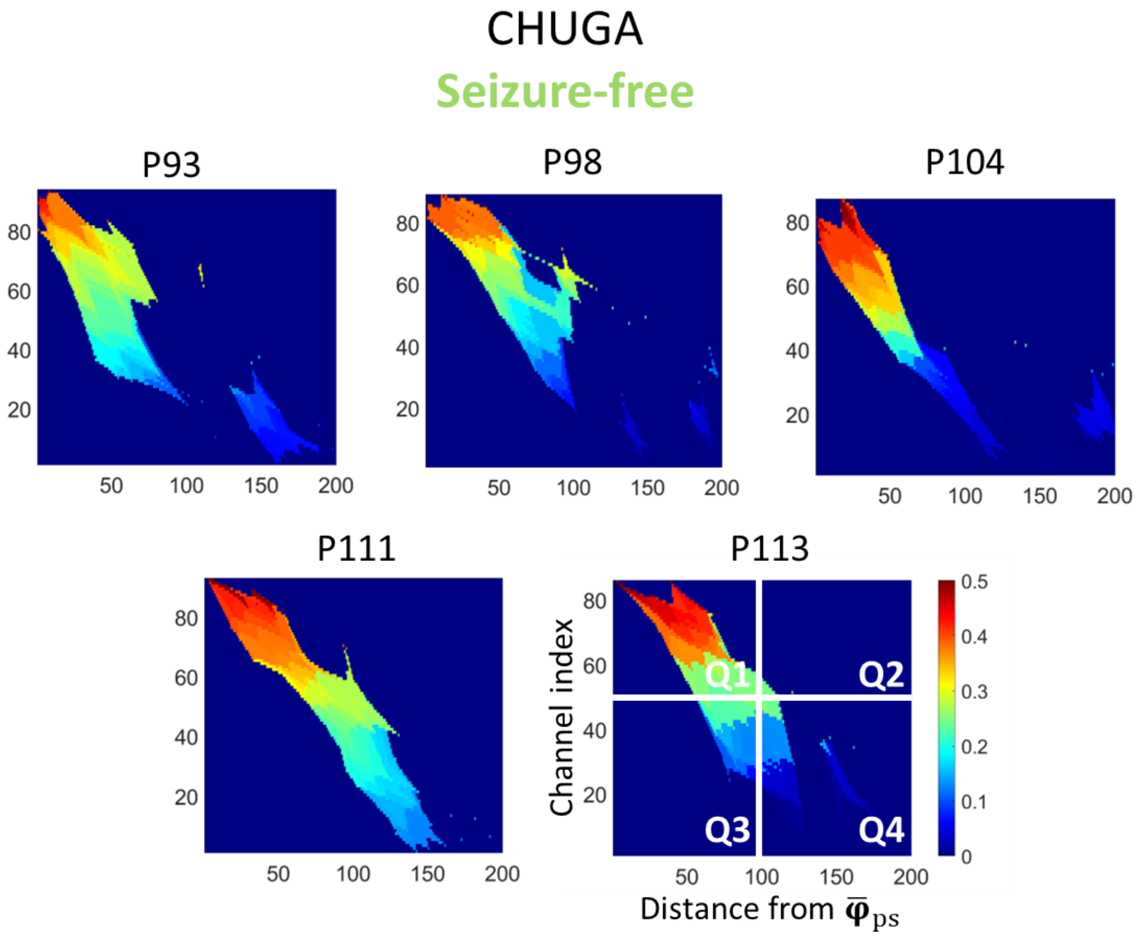

Supplementary Figure S6*: Examples of seizure-free SP maps from the CHUGA patient cohort.* *Five examples of the SP maps computed from seizure-free patients in the CHUGA cohort are shown to demonstrate the consistencies in the patterns described in **Figure 6** of the main manuscript, when applying the method on a different patient cohort extracted from an independent center. Abbreviations: CHUGA= Grenoble Alpes University Hospital Center, Q=Quadrant, SP=Spatial perturbation.*

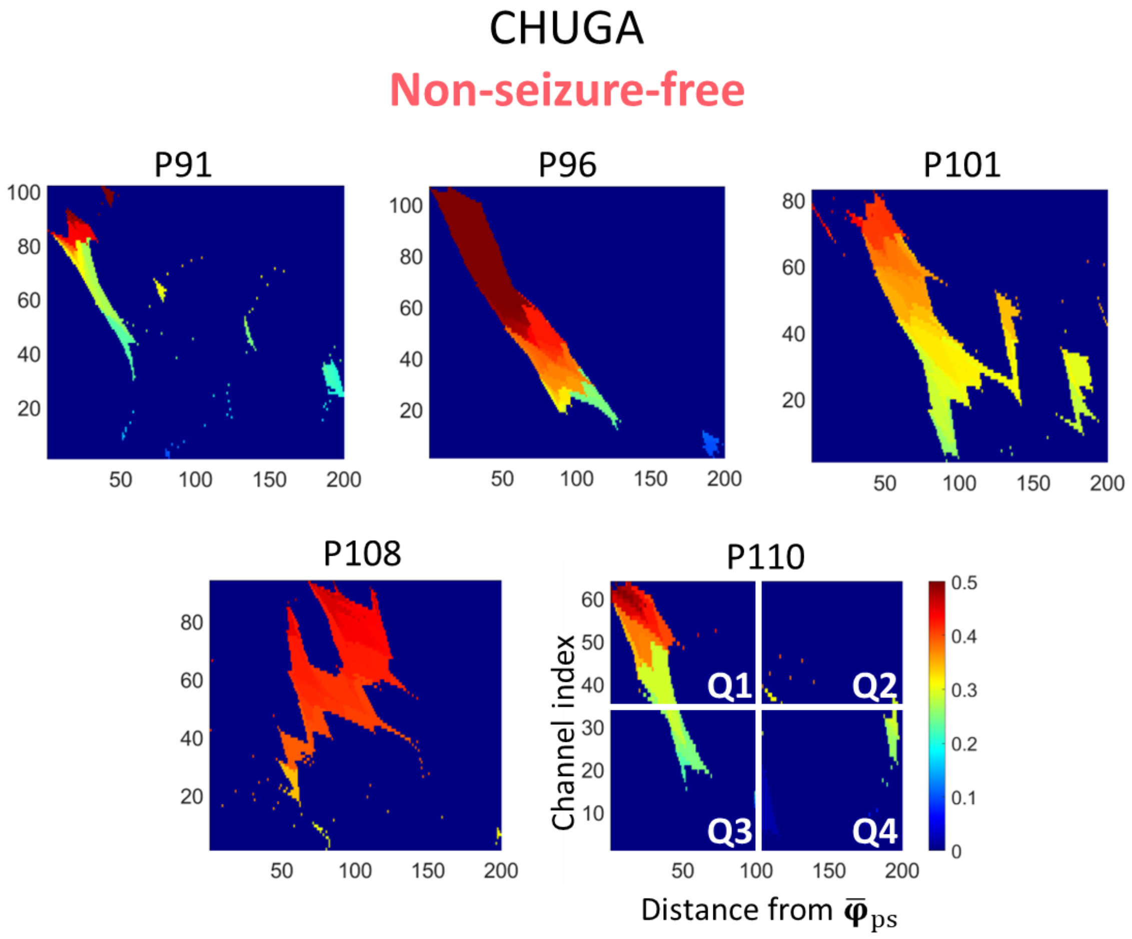

Supplementary Figure S7***: Examples of non-seizure-free SP maps from the CHUGA patient cohort.*** *Five examples of the SP maps computed from non-seizure-free patients in the CHUGA cohort are shown to demonstrate the consistencies in the patterns described in* ***Figure 6*** *of the main manuscript, when applying the method on a different patient cohort extracted from an independent center.*

Abbreviations: CHUGA= Grenoble Alpes University Hospital Center, Q=Quadrant, SP=Spatial perturbation.

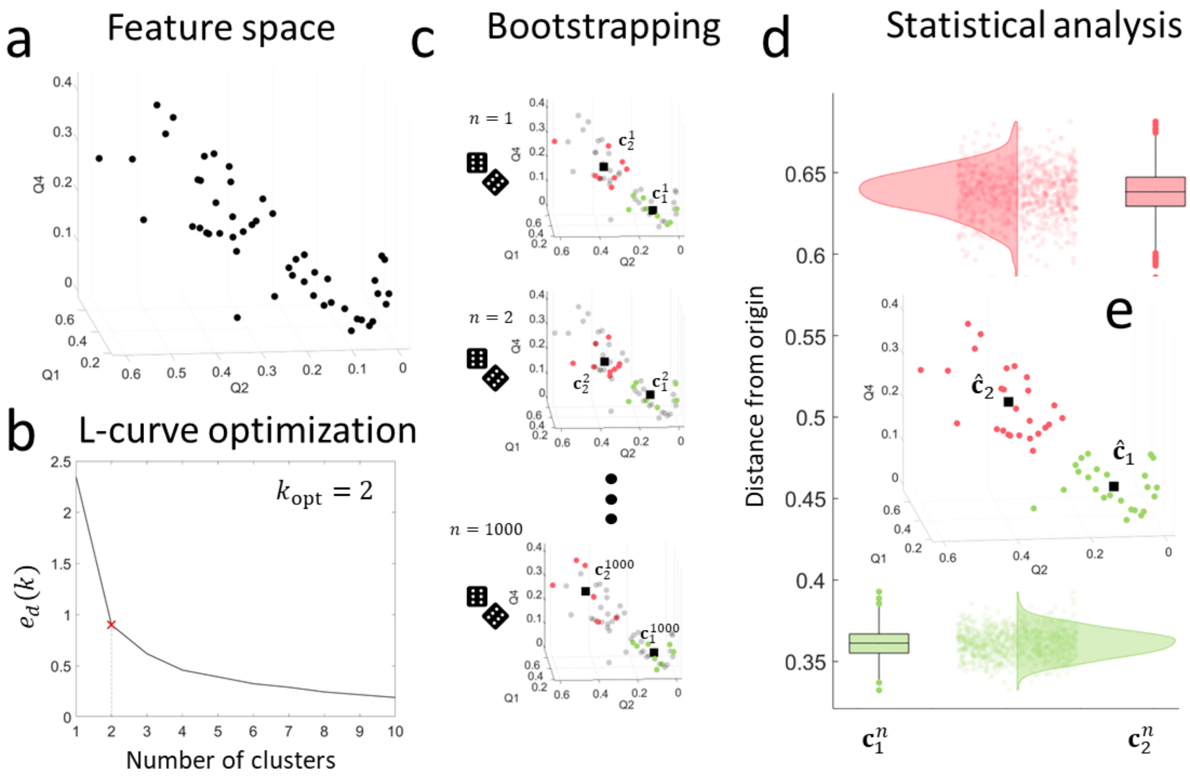

Supplementary Figure S8*: Centroid estimation pipeline.* a *The feature space for the MNI patient cohort. Qualitatively, one can see two distinct clusters.* ***b*** *The L-curve approach was taken to quantitatively find the optimal number of clusters.* ***c*** *The data is then split such that 75% of each class is retained and is randomly sampled 1000 times to generate a total of 1000 centroids.* ***d*** *We see that there is no overlap in the centroids' distance from origin, demonstrating that the clusters are robust.* ***e*** *The median of these centroids is computed to obtain the final centroids* $\hat{\mathbf{c}}_1$ *and* $\hat{\mathbf{c}}_2$ *to reduce outlier effects from the data. Source data are provided as a Source Data file. Abbreviations: MNI=Montreal Neurological Institute.*

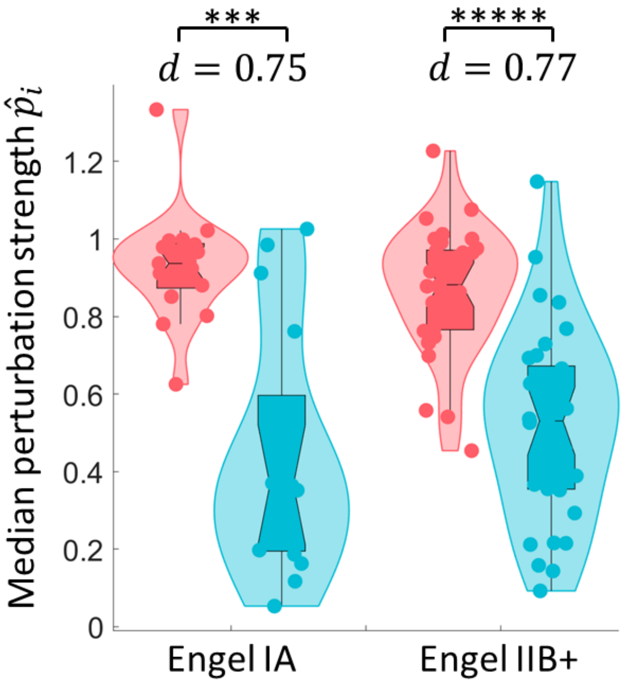

Supplementary Figure S9***: Perturbation strength and association with SOZ.*** *The median of the perturbation strength computed inside (in red) and outside the SOZ (in blue) in the MNI cohort. Each point represents a patient. The median is significantly higher within the SOZ, and therefore, the upper 70th percentile of all perturbation indices was considered when computing the centroid to apply the perturbation in the ranked SP framework. This demonstrates that the perturbation strength is specific to the SOZ (measured by the SEEG) and is not biased towards seizure-free patients. Statistical significance shown in asterisks:* ****p*=2.0×10^{-4} *(n=17; two-sided Wilcoxin rank sum test),* ******p*=7.7×10^{-8} *(n=33; two-sided Wilcoxin rank sum test). Source data are provided as a Source Data file.*

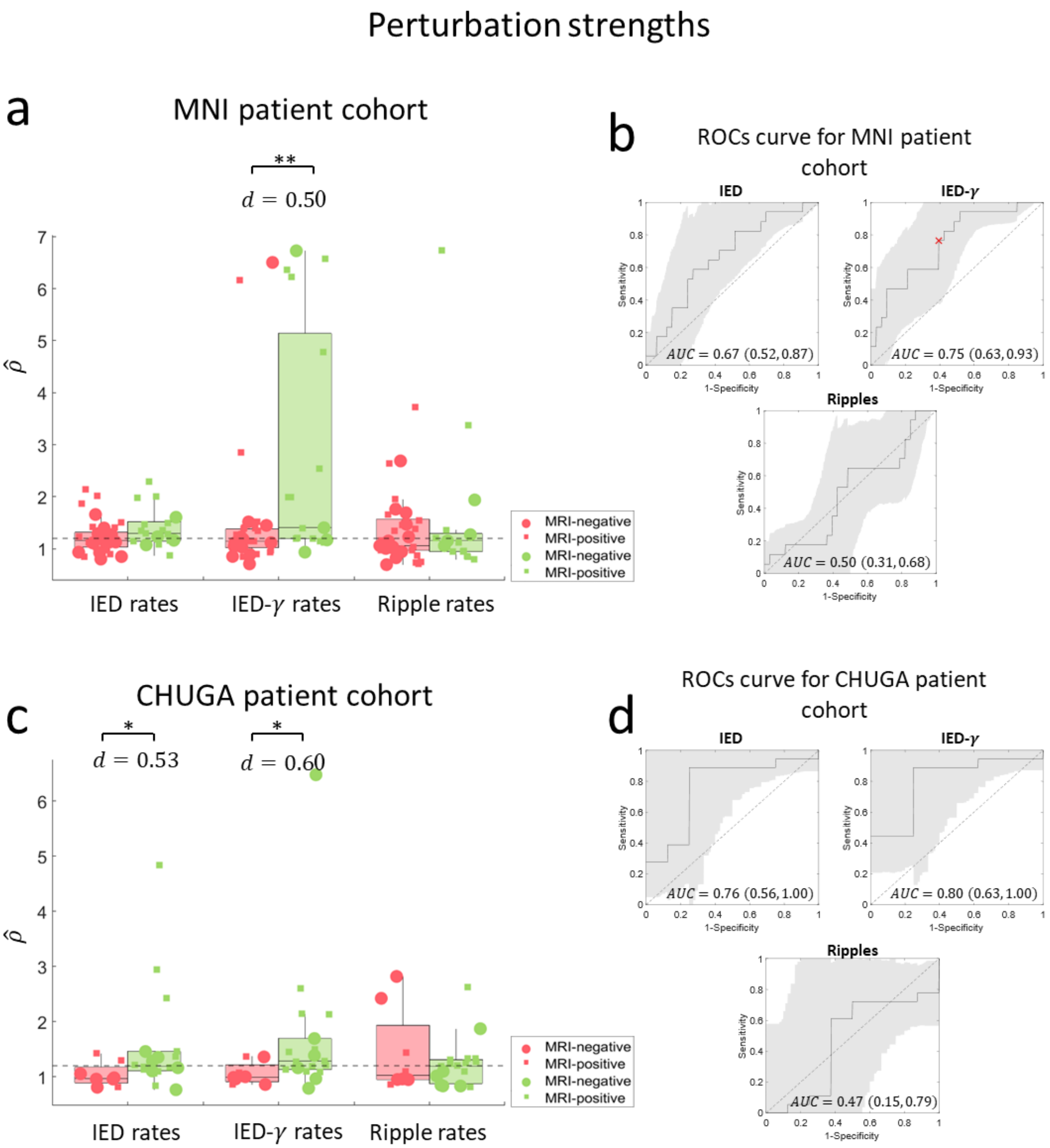

Supplementary Figure S10*: Perturbation strength of IED- γ predicts surgical outcome.* *Comparisons are performed between seizure-free patients (MNI: n=17; CHUGA: n=18) and non-seizure-free patients (MNI: n=33; CHUGA: n=8). The perturbation strength is computed for each patient as well as different interictal EZ biomarkers (described in* ***Methods****).* ***(a)*** *IED rates achieve near significance (*$\hat{\rho}_{\mathrm{SF}}, \hat{\rho}_{\mathrm{non-SF}}$ *=1.30 (0.37), 1.15 (0.29); p=0.054;d=0.34; AUC=0.67 (95% CI: 0.52, 0.87)), IED-γ rates achieve significance with a moderate effect (*$\hat{\rho}_{\mathrm{SF}}, \hat{\rho}_{\mathrm{non-SF}}$ *=1.40 (3.95), 1.14 (0.36); p=4×10*$^{-3}$*; d=0.50; AUC=0.75 (95% CI: (0.63, 0.93)), however, ripple rates do not produce an SOZ network that evaluates the measured SOZ (*$\hat{\rho}_{\mathrm{SF}}, \hat{\rho}_{\mathrm{non-SF}}$ *=1.15 (0.35), 1.06 (0.61); p=0.98; d=0.005; AUC=0.50 (95% CI:0.31, 0.68)).* ***(b)*** *The ROC curves of perturbation strengths using IEDs, IED-γ, and ripples. The threshold used to classify surgical*

outcome is obtained from the ROC curve of the IED-γ spatial system. ***(c)*** *We found the same trends in the CHUGA patient cohort, with IED rates now achieving significance; ripples remained non-significant (**IED**: $\hat{\rho}_{SF}, \hat{\rho}_{non-SF} =$ 1.20 (0.35), 0.97 (0.29);p=0.04; d=0.53; AUC=0.76 (95% CI:0.56, 1.00),* ***IED-γ***: *$\hat{\rho}_{SF}, \hat{\rho}_{non-SF}$ =1.28 (0.56), 0.99 (0.31); p=0.02; d=0.60; AUC=0.80 (95% CI:0.63, 1.00),* ***Ripples***: *$\hat{\rho}_{SF}, \hat{\rho}_{non-SF}$ =1.19 (0.43), 1.03 (0.98); p=0.80; AUC=0.47 (95% CI: 0.15, 0.79)). In both figures, non-SF and SF patients are shown as red and green dots, respectively. MRI-negative patients are represented as a circle, and MRI-positive are presented as a square. Summary statistics are represented as median (IQR). All p-values were computed using a two-sided Wilcoxin rank sum test. $\tilde{\rho}^{*}$ is tested on the CHUGA cohort, obtaining a sensitivity of 0.61 and specificity of 0.75.* ***(d)*** *The ROC curves of the IED, IED-γ and ripple spatial systems in the CHUGA cohort. The threshold is visualized as a horizontal dotted line in* ***(a)*** *and* ***(c)***. *Source data are provided as a Source Data file. Abbreviations: IED=interictal epileptiform discharge, MNI=Montreal Neurological Institute, CHUGA=Grenoble Alpes University Hospital Center, IED=interictal epileptiform discharge, IQR=interquartile range, ROC=receiver operating characteristics. Statistical significance shown in asterisks: *p<0.05, **p<0.01*

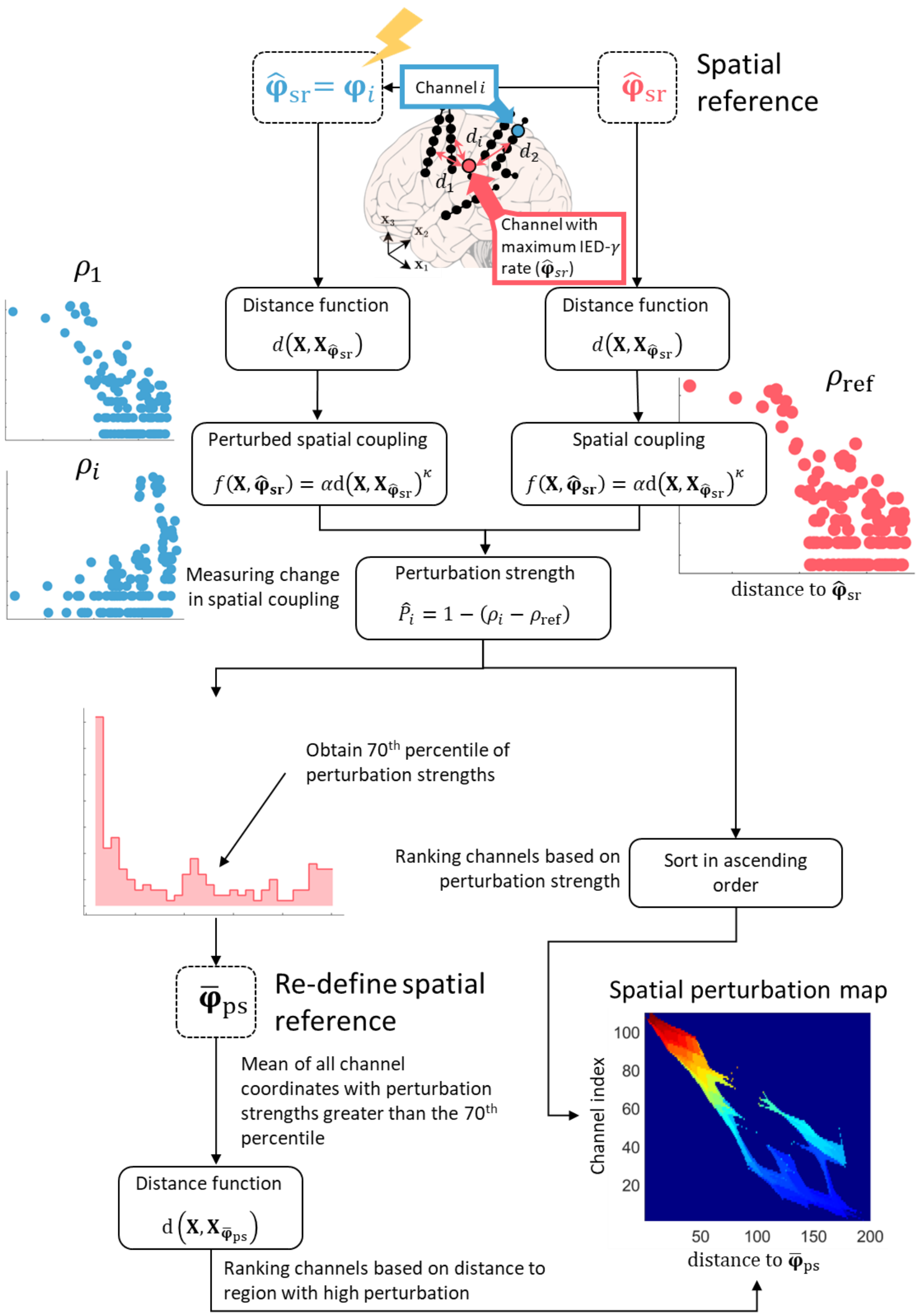

Supplementary Figure S11: ***Detailed flowchart of constructing the SP map.*** *Firstly, a spatial system is constructed using the maximum IED-γ channel as the spatial reference (right column). This spatial system is perturbed by permuting the spatial reference with each channel (left column). The perturbation strength is computed as the change in the spatial system after applying a permutation with channel i. This is done for all channels available in the SEEG implantation scheme, which produces a distribution of perturbation strengths, where each perturbation strength is associated with a channel. A second-step perturbation is applied by redefining the spatial reference as the region of high*

perturbation strength. The spatial reference is computed by averaging the channel coordinates which have perturbation strengths larger than the 70th percentile of the distribution. This is used as the x-axis of the SP map. The channels are sorted in ascending order and plotted in the y-axis. A uniform bin size of 200 is used in the x-axis for uniformity across patients.

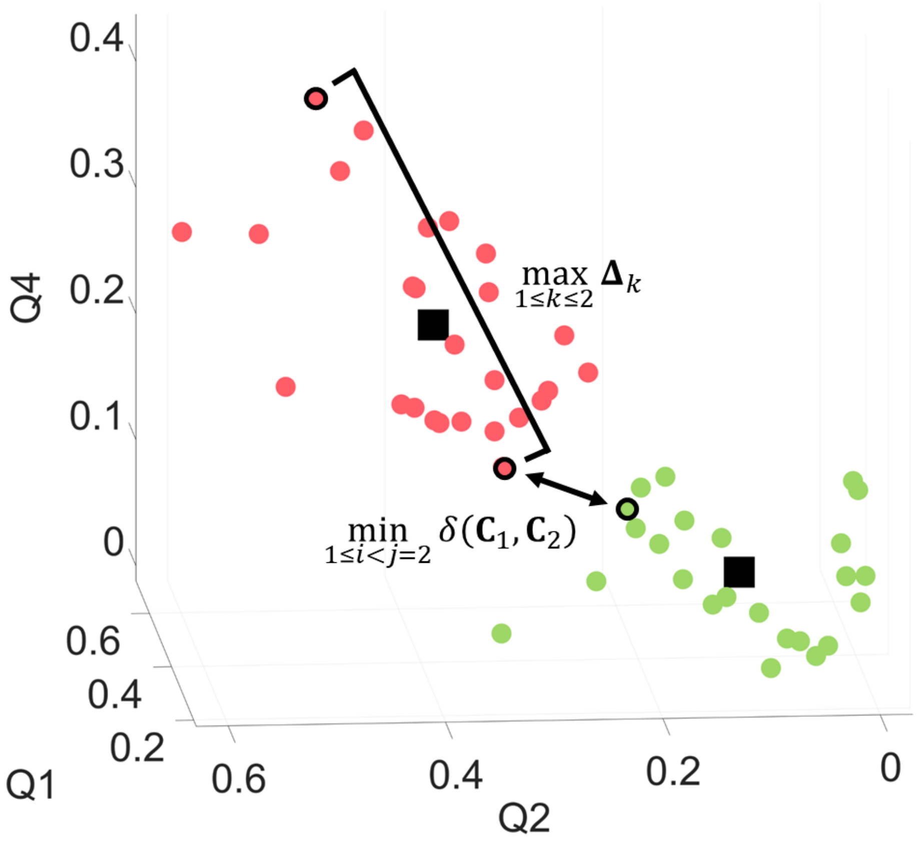

Supplementary Figure S12: ***Calculation of the Dunn's index.*** *An example of calculating the Dunn's index using Q1, Q2 and Q4.* $\max_{1 \le m \le k_{\text{opt}}=2} \mathbf{\Delta}_m$ *calculates the separability between the two clusters, and* $\min_{1 \le i < j = 2} \delta\left(\mathbf{C}_{1,\boldsymbol{F}_{\text{opt}}}, \mathbf{C}_{2,\boldsymbol{F}_{\text{opt}}}\right)$ *calculates the compactness of the clusters. The larger the index, the more compact and separable the clusters. The values itself has no meaning and is only used to decide between the choice of features and the hyperparameters of the pipelines and/or models.*

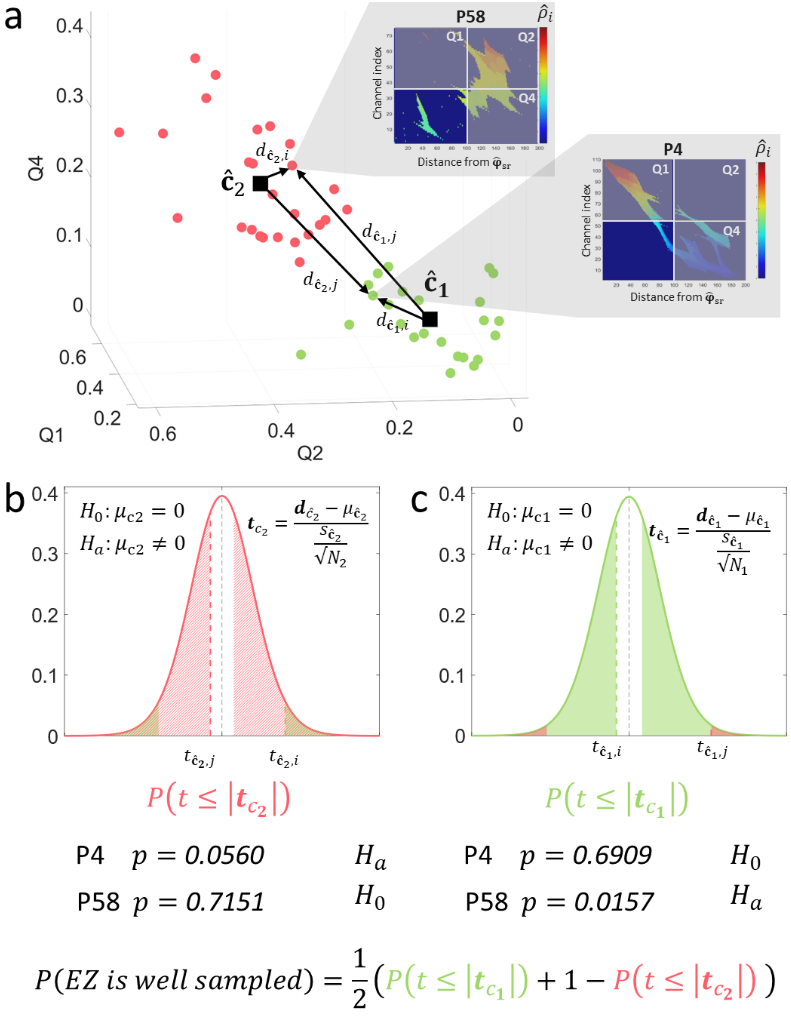

Supplementary Figure S13*: Probability model of SOZ implantation.* *a* *Illustration of the feature space with the MNI patients shown as points. Poorly sampled (red) and well-sampled (green) classification by the k-means algorithm. Examples of patients who are non-seizure-free (P58) and seizure-free (P4) are shown within the feature space as examples for the step-by-step computation of the probabilities.* ***b*** *and* ***c****: A probability distribution is constructed for* $\hat{\mathbf{c}}_2$ ***(b)*** *and* $\hat{\mathbf{c}}_1$ ***(c)*** *by estimating the means and standard deviations of the distances from each centroid for the distances in cluster 1 and 2, respectively. A two tailed hypothesis test is computed for the example patients. P4 rejects the hypothesis that it lies within* $\hat{\mathbf{c}}_2$*, whereas P58 rejects the hypothesis that it lies within* $\hat{\mathbf{c}}_1$*. The final probability is computed as the average probability that the patient lies within* $\hat{\mathbf{c}}_1$*. Abbreviations: EZ=epileptogenic zone.*

TABLES

Table S1: Patient demographics and clinical information

Patient ID	Center	Epileptic focus	Sex	MRI Lesion	Age	DAI (n1, n2)	Engel Class	Number of Electrodes	Electrode Locations	SOZ Channels
P1	MNI	R orbito-frontal, R anterior insula	F	Right orbitofrontal lesion to anterior insula, equivocal of FCD	43	5	IA	7R	RA, RCa, RH, RIa, RIp, ROF, RL	RL1:8, ROF1:8, RIa1:5, RCa1
P2	MNI	R posterior cingulate & precuneus	F	Right posterior cingulate FCD	26	4,6	IA	15R	RA, RCa, RCg, RCi, RCp, RDa, RDb, RDp, RIa, RIp, RLa, RLp, RPc, RSMa, RSMp	RDb1:2, RDa1:5, RDp1:3
P4	MNI	L parietal operculum & supramarginal gyrus	F	FCD Parietal operculum/ inferior parietal lobule FCD (anterior portion of the supramarginal gyrus)	11	6	IA	11L	LCO, LFE, LLea, LLep, LS, LSh, LSMa, LSMs, LSPl, LT1m, LT2p	LLea1:10, LLep1:9
P6	MNI	R temporo-parietal	M	Bilateral Hc atrophy	32	5, 17	IA	11R	RAg, RCu, RFug, RH, RIa, RIp, RLg, RSMa, RSMp, RT1p, RT2p	RFug2:7, RAg1:6, RT2p1:7, RLg8:13, RH8:11, RSMa2:8, RSMp4:14, RIa3:8, RH1:4
P8	MNI	L superior temporal gyrus	M	Postsurgical defect following L mesiotemporal lobe resection, surrounding gliosis and/or encephalomalacia	38	11	IVA	10L	LCp, LFug, LHp, LIa, LIp, LOF, LT1a, LT1p, LT2a, LPc	LT1a1:3
P10	MNI	L temporo-insular (posterior)	F	Left posterior insula and Helsch's gyrus FCD + surgical bed	27	3, 7	III	13L	LCa, LCp, LHa, LHe, LHp, LIa, LIp, LM, LS, LSMA, LSms, LSmi, LpT	LIp4:8, LHe1:5
P13	MNI	L temporo-parieto-occipital	M	Left post quadrant dysplasia, max temporo-occipital (hemihemimegalence-phaly)	45	6, 7	IIB	8L+2R	LA, LCp, LFug, LH, LHp, LOi, LOs, LPC, RA, RH	LHp1:3, LHp5:10, LH1:3, LFug2:7, LCp6:10, LCp13:15, LPC3:11, LOs6:8, LOi5:10
P16	MNI	L mesio-temporal	F	Left post fusiform or para Hc cystic lesion	40	6, 8	IID	7L	LA, LCp, LE, LEa, LHa, LHp, LL	LA1:3, LHa1:3, LHp1:3
P17	MNI	R fronto-temporal (widespread: orbitofrontal, anterior cingulate, anterior insula, pole frontal & mesio-temporal)	F	normal	30	4	IIIA	2L+8R	LCa, LOF, RA, RCa, RCm, RFP, RH, RHp, RL, ROF	RFP6:10, ROF1:4, RL2:7, RCa1:2, RCa8:9, RCm8:10, RA1:4, RH1:2
P18	MNI	R precuneus, R mesial postcentral gyrus, R superior parietal lobule	F	Right pre-cuneus FCD	21	5, 6	IA	5R	RCAg, RCSMg, RLa, RLp, RPs	RLa1:10, RLp1:9, RCAg3:5
P19	MNI	R mesio-temporal	F	Right Hc atrophy	55	3, 13	IIB	3L+3R	LA, LH, LHp, RA, RH, RHp	RA1:2, RH1:2, RHp1:3
P20	MNI	Right middle frontal gyrus	M	Right mid F convexity FCD	36	5	IA	2L+6R	LCa, LCm, RA, RCa, RCm, RH, RLi, RLs	RLi1:7

P22	MNI	L anterior cingulate, L orbito-frontal, L amygdala	M	Left anterior cingulate FCD	37	3, 5	IA	5L+2R	LA, LCa, LCm, LH, LOF, RCa, RH	LCa1:3, LOF1:3, LA1:3
P24	MNI	L mesio-temporal	F	Left Hc atrophy.	31	3, 16	IIB	8L+2R	LA, LCa, LCi, LCp, LFug, LH, LHp, Loi	LFug1:3, LH1:4, LHp1:3, LA1:3
P26	MNI	L mesio-temporal	F	Left PCA remote ischemic lesion + R mirror discreet lesion in PCA	32	4, 13	IIB	8L	LA, LCi, LFug, LHp, LOi, LOs, LPc, LTp	LA4:9, LHp3:9, LFug3:7
P27	MNI	R temporo-insular	F	normal	28	2, 13	IA	7R	RA, RH, RHp, RIa, ROF, RparH, RTa	RTa2:7, RA5:9, RH5:8, RHp6:8, RparH4:9, RIa1:4
P28	MNI	R temporo-insulo-parietal	M	Diffuse atrophy	37	4, 14	IVC	14R	RCa, RCp, RFa, RHe, RHp, RI, RL, RMi, RMs, ROF, RPC, RSMA, RSi, RSs	RHe2:6, RI6:8, RSi1:3
P29	MNI	L middle frontal gyrus	M	Left pre-central, deep (F1 and F2) FCD.	25	3, 5	IA	6L	LCa, LCm, LlF, LMF, LPC, LSMAa	LMF1:6, LlF5:6
P30	MNI	L fronto-temporal (orbito-frontal, anterior cingulate, mesio-lateral temporal)	F	Left ant cingulate/OF FCD.	23	2, 9	IIIA	7L	LA, LCa, LCp, LH, LHp, LOF, LOI	LOF1:3, LOi1:4, LCa1:4, LA1:3, LA7:9, LH1:4, LHp1:4, LHp6:9
P32	MNI	L Hippocampus, posterior cingulate	F	Left MTS; surgical bed.	26	5, 19	IA	6L	LCa, LCp, LH, LHp, LI, LPCu	LCp1:2, LH1:3, LHp1:2
P34	MNI	R Temporal-insula-frontal-parietal	F	normal	32	10, 12	III	7R	RA, RCa, RCp, RH, RHp, Rip, RL	RH6:9, RHp1:3, RCa6:9, RCp6:9, RA7:9, RIp5:7
P35	MNI	L mesio-temporal	F	Left Hc atrophy + signal change	43	5, 15	IVA	4L+4R	LA, LH, LHp, LOF, RA, RH, RHp, ROF	LA1:3, LH1:3, LHp1:3
P36	MNI	R SMA, R mid-cingulate and R mesial superior frontal gyrus	F	Right F1 and F2 FCD	22	12	IIIA	2L+7R	LH, LIp,RCa, RCp, RH, RIp, RSMAa, RSMAm, RSMAp	RSMAa1:7, RSMAm7:9, RSMAp7:9
P37	MNI	R mesio-temporal	F	Right Hc hypersignal (R T hypometabolism on FDG PET); cystic-like lesion, L F.	25	4, 14	IVB	1L+6R	LFL, RA, RCa, RH, RHp, Ria, ROF	RA1:3, RH1:6, RHp1:3, RHp7:10
P38	MNI	R fronto-temporal	M	normal	41	6, 14	IVA	9R	RA, RAC, RH, RHp, RIP, RMC, ROF, RPC, RSMA	RAC10:12, RSMA1:8, RMC8:12, ROF8:12, RH1:3, RHp1:3
P39	MNI	L mesio-temporal	M	normal	25	2, 14	IVB	4L+4R	LA, LH, LHp, LOF, RA, RH, RHp, ROF	LH1:4, LA1:2
P41	MNI	L mesio-temporal	M	normal	33	7, 16	IIIA	4L+4R	LA, LH, LO, LP, RA, RH, RO, RP	LA1:9, LH1:3, LP1:3
P42	MNI	L fronto-parietal	M	normal	18	5	IIIA	5L+5R	LCA, LCP, LOF, LSMAa, LSMAp, RCA, RCP, ROF, RSMAa, RSMAp	LCA7:18, LCP7:18, LSMAa1:4, LSMAa7:18
P43	MNI	R mesial and lateral temporal	F	normal	52	2, 14	IVA	3R	RA, RH, RHp	RHP3:7, RH1:3
P45	MNI	R mesio-temporal	M	normal	53	2, 9	IVB	8R	RA, RCi, RCp, RH, RHp, ROi, ROs, RPc	RA1:3, RHp1:3, RH1:3, RCi1:4
P46	MNI	R fusiform gyrus & R posterior nodule	M	PVNH bilateral occipital horn, Right temporal horn	21	4, 8	IIIA	4L+10R	LHa, LHp, LNa, LNp, RFua, RFup, RHa, RHm, RHp, RNa, RNp, RSM, RT1a, RT1p	RNp1:3, RFua1:5, RFup1:7

				(adjacent to Right hippocampus)						
P49	MNI	B mesio-temporal, R lateral parietal	M	Bilateral PO atrophy and gliosis (precuneus and cuneus); surgical bed.	40	5, 18	III	3L+6R	LCP, LH, LHp, RCA, RH, RHp, ROi, RPI, RPS	LH1:3, LHp1:3, RPS4:6, RCA1:3, RHp1:3
P51	MNI	R fronto-temporal (orbito-frontal, anterior cingulate & mesio-temporal)	M	Normal	36	2, 6	III	7R	RA, RCa, RCp, RH, RHp, RIa, ROF	RA6:8, RH6:8, RHp6:8, ROF6:8, RCa6:8, RIa4:6
P53	MNI	L posterior insula, parietal operculum	M	Right centro-parietal and post insular encephalomalacia	21	2, 7	IIIA	7L	LCAg, LCp, LCSMg, LH, LIa, LIp, LP	LIp1:4, LIa1:2
P56	MNI	L temporo-parieto-occipital	F	Left Hc atrophy; Left fusiform gyrus and pericalcarine atrophy; mild diffuse Left hem atrophy.	37	2, 4	IIIA	9L	LA, LCa, LCp, LFug, LH, LHp, LOi, LOs, LPc	LPc3:6, LCp1:3, LOs2:7, LOi1:3, LFug1:2, LFug6:9, LA1:5, LH1:5, LH8:9, LHp1:2
P58	MNI	L SMA	M	Normal	37	7	IVC	7L+7R	LCa, LCm, LCp, LH, LIi, LOF, LSMAa, RCa, RCm, RCp, RH, RIi, ROF, RSMAa	LSMaA1:3
P59	MNI	L mesio-temporal (Amygdala, Hippocampus, Parahippocampal gyrus)	F	Normal	44	4, 18	IA	8L	LA, LCa, LCp, LH, LHp, LOF, LTa, LTm	LA1:3, LH1:3, LHp1:3, LTm1:3
P60	MNI	L temporo-fronto-parietal	M	Normal	26	6, 15	III	7L	LCa, LCm, LCp, LH, LHp, LI, LOF	LCp1:2, LCp5:9, Li2:6, LHp1:2, LHp4:7, LH5:8
P61	MNI	L temporo-fronto-parietal	M	Normal	36	4, 14	IA	7L (+5L added later!)	LA, LH, LHp, LIS, LOF, RH, RHp, LCa, LCm, LTp, LIp, LPs	LTp1:5, LIp1:2, LOF6:9, LIS1:3, LA1:9, LH1:9, LHp1:9
P62	MNI	L mesio-temporal	F	Normal	38	5, 18	III	4L+2R	LA, LH, LHp, LOF, RA, RH	LA1:5, LH1:3, LHp1:4
P66	MNI	R mesio-temporal	M	Right MTS; Left O uligyria/ encephalomalacia	22	3, 16	IA	2L+5R	LOi, LPs, RA, RH, RHp, ROi, RPs	RA1:3, RH1:4, RHp1:3
P69	MNI	L fronto-insular	M	Normal	36	4, 8	IIIA	9L+2R	LA, LF2a, LFp, LH, LHp, LI, LOF, LSMAa, LSMAp, RCa, RH	LOF1:7, LI1:7
P72	MNI	L temporo-insular (posterior)	F	Left Heschl gyrus and post. insula FCD	53	4, 7	IVB	5L	LA, LH, LHp, LIs, LHe	LHe3:6, LIs3:6
P73	MNI	L middle frontal gyrus	F	Left F (F2) FCD	28	3, 4	IA	5L	LCa, LCm, LFS, LH, LOF	LCm6:9, LFS4:9
P75	MNI	R frontal parasagittal	M	Right F parasagittal FCD	16	3	IA	3R	RC, RL, RSMA	RL1:7
P76	MNI	R orbito-frontal	F	normal	26	3, 11	IA	9R	RA, RCa, RCm, RH, RHp, RIm, ROF, RSMAa, RSMAp	ROF5:10
P78	MNI	R orbitofrontal, R anterior insula, R SMA	M	Right hemi-megalencephaly	33	5, 8	IIIA	9R	RA, RCa, RCm, RH, RIa, RIp, ROF, RSMAa, RSMAp	RIa1:3, ROF9:13, RSMAa1:3
P79	MNI	L mesio-temporal	F	Left ant encephalomalacia; Left Hc atrophy	54	6, 11	IIB	6L	LA, LCa, LCm, LH, LHp, LOF	LH1:3, LHp1:3, LA1:3
P81	MNI	L mid-cingulate and SMA	F	Left mid-cingulate/inf SMA FCD (along calloso-marginal sulcus)	11	6	IA	11L	LCa, LCm, LCp, FMRI, LIa, LIm, LLe, LOF, LSMa, LSMm, LSMp	LSMp1:7, LCm1:4, LSMm1:3

P90	MNI	L Temporal-frontal-parietal	F	Left PMG involving FTP and insular cortex	22	4, 13	IVB	6L	LA, LCa, LH, LHp, LOF, LPi	LOF1:9, LA1:7, LH1:7, LHp1:7, LPi1:3
P91	CHUGA	R Temporal+ temporo-insulo-basal	F	Right Hc atrophy and Right temporal lobe atrophy	50	3	IID	13	d, f, g, h, i, m, n, p, q, r, s, u, v	h13:15, p1:2, g1:3
P92	CHUGA	L TLE	M	normal	43	3	II	16	a_, b_, b, d_, e_, e, f_, f, g_, i_, i, q_, t_, t, u_, u	g_1:10, d_1:10, i_1:8, a_1:14, b_1:14, e_2:13, f_1:13, t_1:7, q_1:2
P93	CHUGA	L TLE	F	normal	30	3	IA	14	a_, b_, c_, e_, f_, g_, i_, l_, o_, p_, t_, u_, v_, y_	a_1:3, b_1:3, c_1:3, e_3:6, f_2:6
P94	CHUGA	R FLE	M	Bifrontal posttraumatic lesions	33	3	IA	18	b, f, f_, g_, h_, i, l, m, n, o, p, q, r, s, u, x, y, z	o1:10, p1:10, q1:10, x1:15, l11:14
P95	CHUGA	R Insular	M	Postsurgical cavity	43	4	IA	15	e, g, h, m, n, o, p, q, r, s, t, u, v, x, y	r1:2
P96	CHUGA	L TLE	F	normal	42	4	IIIA	12	b_, c_, d_, e_, et_, f_, g_, i_, o_, t_, u_, v_	d_7:8, f_1:4, b_1:2, e_1:3, i_1:3, t_1:2, c_1:3, et_1:3
P97	CHUGA	L TLE	F	Left hippocampal and entorhinal cortex FCD	24	3	IA	15	a_, a, b_, b, c_, et_, i_, q_, r_, r, t_, u_, u, x_, x	a_1:3, b_1:4, c_1:3, et_1:5
P98	CHUGA	R TLE	M	normal	52	3	IA	15	a, b, c, d, e, et, f, i, p, s, t, u, v, w, x	a1:4, et1:4, b1:4, c1:4, e1:2
P99	CHUGA	L FLE	M	Left frontobasal scar (resection cavernoma gyrus rectus)	61	1	IA	12	a_, b_, c_, d_, e_, g_, i_, l_, o_, q_, t_, x_	t_1:2, l_2:3, a_1:4, b_1:2, c_1:2, e_1:4
P100	CHUGA	R FLE	M	Right frontal tumor residue	33	3	IA	15 (16)	b, c, e, f, h, i, l, o, q, r, t, u, w, x, y	o1:6, w1:7, f1:3
P101	CHUGA	R TLE	F	normal	15	1	III	18	a, b, b_, e, f, f_, g, h, h_, o, o_, p, t, u, v, v_, x, y	a1:2, b1:3, e1:3
P102	CHUGA	L TLE	F	Left hippocampal sclerosis with bad gray-white matter differentiation and atrophy at the temporal pole	26	1	IA	14	a_, b_, c_, f_, g_, i_, k_, o_, q_, r_, s_, t_, u_, y_	a_1:4, b_1:2, c_1:2
P103	CHUGA	R FLE	F	normal	14	7	IA	22	a, c, d, e, f, g, h2, i, j, k, l, m, n, o, p, s, u, v, w, x, y	l2:3, f6:8, n7:9, k6:8, m6:8, g6:8
P104	CHUGA	R TLE	F	Cystic lesion	36	3	IA	17	a, b, b_, e, et, g, I, o, r, s, s_, t, u, x, x_, y, y_	t1:3, a1:3, b1:4, i4:8, b_1:3, y2:4, et2:3
P105	CHUGA	R mesioFLE	F	Right frontal upper opercular region FCD	19	6	IA	22	b, c, d, e, f, g, h, I, j, l, m, n, o, q, r, s, t, v, w, x, y, z	j1:2, m1:3, m10:11, n1:3, d7:10, c6:10, z2:7, r1:8, q2:6, l1:2
P106	CHUGA	TLE	M	normal	38	2	IA	17	a, b, b_, c, d, e, et, p, r, s, t, u, v, v_, w, y, y_	t1:5, w6:8, y1:2
P107	CHUGA	L FLE	M	normal	41	11	IA	15	a_, b_, f_, g_, h_, i_, l_, m_, o_, q_, s_, t_, x_, y_, z_	l_1:3, f_2:3, h_1:3, q_1:3, o_13:14, m_1:2
P108	CHUGA	R Fronto-insular	F	normal	26	1	IVB	18	a, e, f, g, h, i, l, m, n, o, p, q, r, s, t, x, y, z	y1:12, m1:6, a1:3, a5:7, e1:2, f1:2, f5:6, h1:2, l1:2, x12:15, z1:3
P109	CHUGA	R FLE	M	normal	21	5	IVA	14	a, b, h, i, j, k, l, m, n, o, p, v, w, y	n1:5, m1:11, l1:2, l7:8, b1:8, w1:8, h1:12, a1:4, y7:8, k1:5

P110	CHUGA	R parietal	F	Multiple tubers and subependymal nodules	6	6	IIB	14	b, d, e, f, h, i, j, k, l, n, o, p, s, w	b1:12, h1:18, o1:10, s1:18, l2:9, p2:8, k10:11
P111	CHUGA	L TLE	F	Discreetly thickened cortex at the Left upper temporal gyrus + temporal-insulin-opercular junction	8	2	IA	11	b_, c_, i_, l_, m_, p_, r_, s_, t_, u_, v_	m_1:3, t_1:6, u_1:2
P112	CHUGA	R TLE	M	Right Hc sclerosis	47	1	IIIA	17	b, c, d, e, et, f, g, i, o, q, r, t, u, v, x, y, z	i1:2, e1:3, b1:3, x1:2
P113	CHUGA	L TLE	M	Temporal pole and mesial structural FCD	13	9	IA	15	a_, b_, c_, d_, e_, f_, i_, q_, r_, s_, t_, u_, v_, w_, x_, y_	i_1:4, a_1:2, b_1:3, e_2:6, c_1:3, x_1:2
P114	CHUGA	R TLE	F	Left intraparietal sulcus signal anomaly; R Hc atrophy	41	2	IA	11	b, b_, c, c_, d, e, f, i, t, u, v	i1:4, b1:5, c1:2
P115	CHUGA	L TLE	F	normal	54	1	IA	12	a_,b_,c_,et_,e_,f_,i_,t_,u_,s_,v_,y_	i_1:6, b_1:3, a_1:3, c_1:3, e_1:4, et_1:5
P116	CHUGA	R Insular	M	Right insulo-opercular region FCD	7	3	IA	15	a, b, e, f, i, j, p, q, r, s, t, u, v, y, z	i5:7, p1:2

Abbreviations: a = anterior; Ag = angular gyrus; A = amygdala; b = body; C = cingulate gyrus; Cge = cingulate gyrus genu; Cis = isthmus of the cingulate gyrus; Cun = cuneus; DAI = day after implantation; Ec = entorhinal cortex; F = female; FCD = focal cortical dysplasia; FO = frontal operculum; FP = frontal pole; Fus = fusiform gyrus; g = gyrus; H = hippocampus; He = Heschl gyrus; I = insula; i = inferior; L = left; Le = lesion; LL = lingual gyrus; M = male; m = middle; M = motor cortex; n1 = night #1; n2 = night #2; NH = nodular heterotopia; O = occipital; OF = orbito-frontal; P = parietal; PC = precuneus; PH = parahippocampus; p = posterior; R = right; S = sensory cortex; SM = supra-marginal gyrus; SMA = supplementary motor area; s = superior; TO = temporal operculum; TP = temporal, MNI=Montreal Neurological Institute, CHUGA= Grenoble Alpes University Hospital Center

Table S2: 'True' classification performance of clustering for different thresholds

Threshold	Resected SOZ Volume (cm^3)	MNI			CHUGA		
		$n_{\mathrm{SF}}, n_{\mathrm{non-SF}}$	Sensitivity	Specificity	$n_{\mathrm{SF}}, n_{\mathrm{non-SF}}$	Sensitivity	Specificity
70th	3.134	11,10	0.73	0.90	9,4	0.67	1.00
75th	3.305	10,9	0.70	0.89	8,4	0.75	1.00
80th	3.557	8,7	0.63	1.00	5,4	0.80	1.00
85th	3.800	8,6	0.63	1.00	5,4	0.80	1.00

Abbreviations: AUC=area under the curve; CHUGA= Centre Hospitalier Universitaire de Grenoble; MNI=Montreal Neurological Institute; SF=seizure-free; SOZ=seizure-onset zone

Table S3: 'True' classification performance of probability model for different thresholds

Threshold	Resected SOZ Volume (cm^3)	MNI				CHUGA			
		$n_{\mathrm{SF}}, n_{\mathrm{non-SF}}$	p value	Cliff's d	AUC	$n_{\mathrm{SF}}, n_{\mathrm{non-SF}}$	p value	Cliff's d	AUC
70th	3.134	11,10	1.0×10^{-3}	0.84	0.92	9,4	0.15	0.56	0.78
75th	3.305	10,9	2.0×10^{-3}	0.82	0.91	8,4	0.11	0.63	0.81
80th	3.557	8,7	6.2×10^{-4}	0.96	0.98	5,4	0.06	0.80	0.90
85th	3.800	8,6	1.0×10^{-3}	0.96	0.98	5,4	0.06	0.80	0.90

Abbreviations: AUC=area under the curve; CHUGA= Centre Hospitalier Universitaire de Grenoble; MNI=Montreal Neurological Institute; SF=seizure-free; SOZ=seizure-onset zone

Table S4: Dunn's index for different feature combinations[1]

Feature combinations	Number of clusters								
	2	3	4	5	6	7	8	9	10
Q34	0.055	0.113	0.036	0.090	0.097	0.085	0.149	0.082	0.172
Q24	0.204	0.095	0.099	0.139	0.099	0.099	0.142	0.131	0.123
Q23	0.089	0.107	0.108	0.080	0.107	0.123	0.110	0.090	0.102
Q14	0.073	0.081	0.108	0.108	0.146	0.072	0.128	0.153	0.134
Q13	0.064	0.139	0.112	0.138	0.025	0.208	0.031	0.061	0.085
Q12	0.053	0.100	0.088	**0.156**	0.051	0.105	0.074	0.143	**0.188**
Q234	0.159	0.159	0.061	0.228	0.123	0.090	0.151	0.141	0.182

[1] The bold text in **Table S4** represents the maximum Dunn's index for each column.

Q134	0.078	0.078	0.156	0.077	0.153	0.105	0.165	0.081	0.054
**Q124**	**0.255**	**0.255**	**0.309**	0.096	**0.158**	**0.215**	0.181	0.173	0.154
Q123	0.096	0.096	0.107	0.084	0.088	0.132	0.181	0.182	**0.188**
Q1234	0.191	0.191	0.168	0.113	0.101	0.146	**0.263**	**0.214**	0.185
Argmax	**Q124**	**Q124**	**Q124**	Q12	**Q124**	**Q124**	Q1234	Q1234	Q123

Supplementary Pseudocode

Algorithm 1 Virtual-removal spatial perturbation framework

Input Feature vector $\boldsymbol{f} \in \mathbb{R}^{N\times M}$, channel coordinates $\boldsymbol{X} \in \mathbb{R}^{N\times 3}$, boolean array $isSoz \in \{0,1\}^{N\times 1}$

Output Perturbation strength $\hat{\rho}$

for each segment $j \in \{1,2,\ldots,m\}$ such that $\max\big(f(:,j)\big) > 1$

  get $\boldsymbol{f}_s \leftarrow \boldsymbol{f}(:,j)$

  // Step 1: Construct and characterize spatial system

  compute spatial reference index $\hat{k} \leftarrow \underset{isSOZ(k)==1}{\operatorname{argmax}} \{\boldsymbol{f}_s\}$ // Max Channel in SOZ

  compute distance $\boldsymbol{d} \leftarrow \left|\left|\boldsymbol{X} - \boldsymbol{X}(\hat{k})\right|\right|_2$

  //Note: the spatial reference is removed prior to computing the correlation in $corr$

  characterize spatial system $\rho_{BR}^{j} \leftarrow corr(\log(\boldsymbol{f}_s + 0.1), \log(\boldsymbol{d} + 0.1))$ // Pearson's correlation.

  // Step 2: Virtually remove the seizure-onset zone

  remove $\boldsymbol{f}_s(isSOZ)$, $\boldsymbol{d}(isSOZ)$, $\boldsymbol{X}(isSOZ,:)$

  re-compute spatial reference index $\hat{k} \leftarrow \underset{k}{\operatorname{argmax}}(\boldsymbol{f}_s)$

  re-compute distance $d \leftarrow \left|\left|\boldsymbol{X} - \boldsymbol{X}(\hat{k})\right|\right|_2$

  if $\max(\boldsymbol{f}_s) > 1$ // Set correlation to zero as it's noisy

    characterize spatial system $\rho_{AR}^{j} \leftarrow corr(\log(\boldsymbol{f}_s + 0.1), \log(\boldsymbol{d} + 0.1))$

  else

    set $\rho_{AR} \leftarrow 0$

  end if

end for

get $\bar{\rho}_{BR} \leftarrow \operatorname{median}\left(\rho_{BR}^{j}\right)$

get $\bar{\rho}_{AR} \leftarrow \operatorname{median}\left(\rho_{AR}^{j}\right)$

// Step 3: Compute perturbation strength

get $\hat{\rho} \leftarrow \log\left(\nu + \left|\frac{\bar{\rho}_{BR}}{\bar{\rho}_{AR}}\right|\right)$ for any $\nu > 0$ //$\nu = 2$ for this manuscript

return $\hat{\rho}$

Algorithm 2 Computing the spatial perturbation map

Input Feature vector $\boldsymbol{f} \in \mathbb{R}^{N\times 1}$ and channel coordinates $\boldsymbol{X} \in \mathbb{R}^{N\times 3}$

Output Perturbation strengths $\widehat{\boldsymbol{P}} \in \mathbb{R}^{N\times 200}$

for each segment $j \in \{1,2,\dots,m\}$

  get $\boldsymbol{f}_s \leftarrow \boldsymbol{f}(:,j)$ // Getting segment data

  // Step 1: Construct and characterize spatial system

  compute spatial reference index $\hat{k} \leftarrow \underset{k}{\operatorname{argmax}}(\boldsymbol{f}_s)$

  compute distance $\boldsymbol{d} \leftarrow \left|\left|\boldsymbol{X} - \boldsymbol{X}(\hat{k})\right|\right|_2$

  //Note: the spatial reference is removed prior to computing the correlation in $corr$

  characterize spatial system $\rho_{ref} \leftarrow corr(\log(\boldsymbol{f}_s + 0.1), \log(\boldsymbol{d} + 0.1))$ // Pearson's correlation.

  // Remove the spatial reference from further analysis, we want to see the deviation of the permuted spatial references from the initial once

  remove spatial reference from inputs $\boldsymbol{f}_s(\hat{k})$, $\boldsymbol{X}(\hat{k})$

  // Step 2: Permuting spatial reference and characterizing perturbed spatial system

  for each channel $c \in \{1,2,\dots,N\}\backslash\{\hat{k}\}$

    permute spatial reference index $\hat{k} \leftarrow c$

    re-compute distance $\boldsymbol{d} \leftarrow \left|\left|\boldsymbol{X} - \boldsymbol{X}(c)\right|\right|_2$

    characterize spatial system $\rho_i^j \leftarrow corr(\log(\boldsymbol{f}_s + 0.1), \log(\boldsymbol{d} + 0.1))$

  end if

end for

get $\bar{\rho}_i \leftarrow \operatorname{median}(\rho_i^j)_j$ // Median is applied over all j

compute perturbation strengths $\hat{p}_i \leftarrow \left|1 - (\bar{\rho}_i - \rho_{ref})\right|$

// Step 3: Spatially ranking $\widehat{\rho}_i$ (i.e., second-step perturbation)

$\rho_{70} \leftarrow prctile(\widehat{\boldsymbol{\rho}}, 70)$ // Obtained 70th percentile of all perturbation strengths

delineate region of high perturbation strength $X_{70} \leftarrow \{X_i \in \mathbb{R}^{1x3} \mid \hat{p}_i \geq \rho_{70}\}$

compute new spatial reference coordinate $\overline{\boldsymbol{X}} \leftarrow \operatorname{mean}(X_{70})$

sort perturbation strengths $\widehat{\boldsymbol{\rho}}$

compute distance $\boldsymbol{d} \leftarrow \left|\left|\boldsymbol{X} - \overline{\boldsymbol{X}}\right|\right|_2$

discretize d into 200 bins $\tilde{d} \leftarrow discretize(\boldsymbol{d}, 200)$

construct spatial perturbation map $\widehat{\boldsymbol{P}} \leftarrow \langle \widehat{\boldsymbol{\rho}}, \boldsymbol{d} \rangle$

convolve $\widehat{\boldsymbol{P}}$ with a 20-pixel line structuring element ranging between 10 and 80 degrees

return $\widehat{\boldsymbol{P}}$

Supplementary References

1 Jin, B., So, N. K. & Wang, S. Advances of Intracranial Electroencephalography in Localizing the Epileptogenic Zone. *Neuroscience Bulletin* **32**, 493-500 (2016).

2 Frauscher, B. Localizing the epileptogenic zone. *Current Opinion in Neurology* **33**, 198-206 (2020).

3 Thomas, J. *et al.* A Subpopulation of Spikes Predicts Successful Epilepsy Surgery Outcome. *Ann Neurol* **93**, 522-535 (2023).

4 Janca, R. *et al.* Detection of interictal epileptiform discharges using signal envelope distribution modelling: application to epileptic and non-epileptic intracranial recordings. *Brain Topogr* **28**, 172-183 (2015). https://doi.org:10.1007/s10548-014-0379-1

5 von Ellenrieder, N., Andrade-Valença, L. P., Dubeau, F. & Gotman, J. Automatic detection of fast oscillations (40–200Hz) in scalp EEG recordings. *Clinical Neurophysiology* **123**, 670-680 (2012). https://doi.org:https://doi.org/10.1016/j.clinph.2011.07.050

6 Ho, A. *et al.* Rapid eye movement sleep affects interictal epileptic activity differently in mesiotemporal and neocortical areas [Manuscript submitted for publication]. (2023).

7 Klimes, P., Peter-Derex, L., Hall, J., Dubeau, F. & Frauscher, B. Spatio-temporal spike dynamics predict surgical outcome in adult focal epilepsy. *Clin Neurophysiol* **134**, 88-99 (2022).

8 Dunn, J. C. A fuzzy relative of the ISODATA process and its use in detecting compact well-separated clusters. (1973).

9 Kaplan, D. (ed knee_pt) (MATLAB Central File Exchange, 2023).

10 Tibshirani, R., Walther, G. & Hastie, T. Estimating the number of clusters in a data set via the gap statistic. *Journal of the Royal Statistical Society: Series B (Statistical Methodology)* **63**, 411-423 (2001).

11 von Ellenrieder, N., Peter-Derex, L., Gotman, J. & Frauscher, B. SleepSEEG: automatic sleep scoring using intracranial EEG recordings only. *J Neural Eng* **19** (2022). https://doi.org:10.1088/1741-2552/ac6829

12 Ren, L. *et al.* Gamma oscillations precede interictal epileptiform spikes in the seizure onset zone. *Neurology* **84**, 602-608 (2015).

13 Zelmann, R., Frauscher, B., Phellan Aro, R., Gueziri, H.-E. & Collins, D. L. SEEGAtlas: A framework for the identification and classification of depth electrodes using clinical images. *Journal of Neural Engineering* (2023).
