# Supplementary material for: A spatial perturbation framework to validate implantation of the epileptogenic zone
Source: Nat Commun. 2024 Jun 19;15:5253. doi: 10.1038/s41467-024-49470-z (PMC11187199; doi:10.1038/s41467-024-49470-z)
Supplement: Supplementary file 3 — Reporting Summary [file 41467_2024_49470_MOESM3_ESM.pdf]

Corresponding author(s): Birgit Frauscher

Last updated by author(s): May 22, 2024

Reporting Summary

Nature Portfolio wishes to improve the reproducibility of the work that we publish. This form provides structure for consistency and transparency in reporting. For further information on Nature Portfolio policies, see our Editorial Policies and the Editorial Policy Checklist.

Statistics

For all statistical analyses, confirm that the following items are present in the figure legend, table legend, main text, or Methods section.

n/a	Confirmed	
☐	☒	The exact sample size (*n*) for each experimental group/condition, given as a discrete number and unit of measurement
☐	☒	A statement on whether measurements were taken from distinct samples or whether the same sample was measured repeatedly
☐	☒	The statistical test(s) used AND whether they are one- or two-sided<br>*Only common tests should be described solely by name; describe more complex techniques in the Methods section.*
☒	☐	A description of all covariates tested
☐	☒	A description of any assumptions or corrections, such as tests of normality and adjustment for multiple comparisons
☐	☒	A full description of the statistical parameters including central tendency (e.g. means) or other basic estimates (e.g. regression coefficient) AND variation (e.g. standard deviation) or associated estimates of uncertainty (e.g. confidence intervals)
☐	☒	For null hypothesis testing, the test statistic (e.g. *F*, *t*, *r*) with confidence intervals, effect sizes, degrees of freedom and *P* value noted<br>*Give P values as exact values whenever suitable.*
☒	☐	For Bayesian analysis, information on the choice of priors and Markov chain Monte Carlo settings
☒	☐	For hierarchical and complex designs, identification of the appropriate level for tests and full reporting of outcomes
☐	☒	Estimates of effect sizes (e.g. Cohen's *d*, Pearson's *r*), indicating how they were calculated

Our web collection on statistics for biologists contains articles on many of the points above.

Software and code

Policy information about availability of computer code

Data collection	All recordings at MNI were performed with either Harmonie or Nihon-Kohden EEG systems. Recordings at CHUGA were performed with the Micromed EEG system.
Data analysis	Dunn's index was computed using a third-party MATLAB code (https://www.mathworks.com/matlabcentral/fileexchange/27859-dunn-s-index). The knee point of the L-curve was computed using a third-party MATLAB code as well (https://www.mathworks.com/matlabcentral/fileexchange/35094-knee-point). All data analyses were performed using MATLAB R2023a. The main code is available in the following link: https://doi.org/10.5281/zenodo.11237651

For manuscripts utilizing custom algorithms or software that are central to the research but not yet described in published literature, software must be made available to editors and reviewers. We strongly encourage code deposition in a community repository (e.g. GitHub). See the Nature Portfolio guidelines for submitting code & software for further information.

Data

Policy information about availability of data

All manuscripts must include a data availability statement. This statement should provide the following information, where applicable:

- Accession codes, unique identifiers, or web links for publicly available datasets
- A description of any restrictions on data availability
- For clinical datasets or third party data, please ensure that the statement adheres to our policy

The data that support the findings of this study are available upon request and if in accordance with the respective research ethics boards' policies.

Research involving human participants, their data, or biological material

Policy information about studies with human participants or human data. See also policy information about sex, gender (identity/presentation), and sexual orientation and race, ethnicity and racism.

Reporting on sex and gender	The MNI cohort comprised 54% females (mean age 32±11). The CHUGA cohort comprised 54% females (mean age 32±16). Sex- and gender-based analyses was not included in the study design, as they have not been shown to impact the outcome of epilepsy surgery (Alim-Marvasti et al., 2022; Yun et al., 2006; Krucoff et al., 2017).
Reporting on race, ethnicity, or other socially relevant groupings	Participants were recruited from Montreal (Canada) or Grenoble (France).
Population characteristics	Participants were prospectively recruited from patients with drug-resistant epilepsy undergoing pre-surgical evaluations with stereo-electroencephalography electrodes at the MNI or CHUGA sites for clinical purposes. All data were collected during clinical routine and only involved procedures that were part of routine clinical care.
Recruitment	All consecutive patients who were offered these procedures were invited to participate in this study.
Ethics oversight	This study was approved by the respective research ethics boards (MNI REB IRB00010120, Cogepistim MR004 11.05.21 DRCI CHUGA). Written informed consent was obtained from all patients.

Note that full information on the approval of the study protocol must also be provided in the manuscript.

Field-specific reporting

Please select the one below that is the best fit for your research. If you are not sure, read the appropriate sections before making your selection.

☒ Life sciences ☐ Behavioural & social sciences ☐ Ecological, evolutionary & environmental sciences

For a reference copy of the document with all sections, see nature.com/documents/nr-reporting-summary-flat.pdf

Life sciences study design

All studies must disclose on these points even when the disclosure is negative.

Sample size	Montreal Neurological Institute (MNI) (50 patients; 17 Engel IA, 33 Engel IIB+) and Grenoble Alpes University Hospital Center (CHUGA) (26 patients; 18 Engel IA, 8 Engel IIB+)
Data exclusions	We excluded Engel IB-IIA patients a priori, as we considered them to be ambiguous cases for evaluating the electrode implantation
Replication	Bootstrapping was performed to test test whether the decrease in correlation by virtually removing the measured SOZ is related to the measured SOZ, and not due to a decrease in sample size. It was also used to obtain the confidence intervals for the area under the curves, as well as for estimating the cluster centroids.
Randomization	Not applicable due to the clinical basis of the participant pool.
Blinding	Not applicable due to the clinical basis of the participant pool.

Reporting for specific materials, systems and methods

We require information from authors about some types of materials, experimental systems and methods used in many studies. Here, indicate whether each material, system or method listed is relevant to your study. If you are not sure if a list item applies to your research, read the appropriate section before selecting a response.

Materials & experimental systems

n/a	Involved in the study
☒	☐ Antibodies
☒	☐ Eukaryotic cell lines
☒	☐ Palaeontology and archaeology
☒	☐ Animals and other organisms
☐	☒ Clinical data
☒	☐ Dual use research of concern
☒	☐ Plants

Methods

n/a	Involved in the study
☒	☐ ChIP-seq
☒	☐ Flow cytometry
☒	☐ MRI-based neuroimaging

Clinical data

Policy information about clinical studies

All manuscripts should comply with the ICMJE guidelines for publication of clinical research and a completed CONSORT checklist must be included with all submissions.

Clinical trial registration: This is not a clinical trial according to the NIH definition, therefore, this study was not registered.

Study protocol: Secondary use of data collected in routine clinical care.

Data collection: All data was collected in routine clinical care. There was no study-related procedures. Informed consent was obtained from all patients for secondary data use.

Outcomes: This was a non-interventional study.

Plants

Seed stocks: *Report on the source of all seed stocks or other plant material used. If applicable, state the seed stock centre and catalogue number. If plant specimens were collected from the field, describe the collection location, date and sampling procedures.*

Novel plant genotypes: *Describe the methods by which all novel plant genotypes were produced. This includes those generated by transgenic approaches, gene editing, chemical/radiation-based mutagenesis and hybridization. For transgenic lines, describe the transformation method, the number of independent lines analyzed and the generation upon which experiments were performed. For gene-edited lines, describe the editor used, the endogenous sequence targeted for editing, the targeting guide RNA sequence (if applicable) and how the editor was applied.*

Authentication: *Describe any authentication procedures for each seed stock used or novel genotype generated. Describe any experiments used to assess the effect of a mutation and, where applicable, how potential secondary effects (e.g. second site T-DNA insertions, mosiacism, off-target gene editing) were examined.*
